# Synthesis and Nanoarchitectonics of Novel Squaraine Derivatives for Organic Photovoltaic Devices

**DOI:** 10.3390/nano12071206

**Published:** 2022-04-04

**Authors:** Dragana Vuk, Floren Radovanović-Perić, Vilko Mandić, Vilma Lovrinčević, Thomas Rath, Ivana Panžić, Jerome Le-Cunff

**Affiliations:** 1 Department of Organic Chemistry, Faculty of Chemical Engineering and Technology, University of Zagreb, Marulićev trg 20, 10000 Zagreb, Croatia; vlovrince@fkit.hr; 2 Department of Inorganic Chemical Technology and Non-Metals, Faculty of Chemical Engineering and Technology, University of Zagreb, Marulićev trg 20, 10000 Zagreb, Croatia; fradovano@fkit.hr (F.R.-P.); ipanzic@fkit.hr (I.P.); 3 Institute for Chemistry and Technology of Materials, NAWI Graz, Graz University of Technology, Stremayrgasse 9, 8010 Graz, Austria; thomas.rath@tugraz.at; 4 Xellia Ltd., Slavonska Avenija bb, 10000 Zagreb, Croatia; jerome.le-cunff@xellia.com

**Keywords:** squaraines, alkyl/aryl aminosquaraine, amination synthesis, hybrid–organic photovoltaics, bulk heterojunction

## Abstract

Necessary advancements in the area of organic photovoltaic (OPV) devices include the upgrade of power conversion efficiencies (PCE) and stability. One answer to these demands lies in the research into new absorbers. Here, we focus on the development of new small molecule absorbers from the group of squaraines (SQs). These modular absorbers can be applied as donors in organic solar cells and have the ability to utilize a broad range of solar radiation if blended with suitable acceptors. In order to allow for the compatibility and favorable organization of donor and acceptor in the absorber layer, we intend to optimize the structure of the SQ by varying the groups attached to the squaric acid core. For that purpose, we accordingly developed a well-suited synthesis route. The novel alkyl- and benzyl-substituted aryl aminosquaraines were synthesized through an improved and eco-friendly procedure. Special emphasis was placed on optimizing the amination reaction to obtain initial precursors in the synthesis of squaraine, avoiding hitherto common catalytic processes. All SQ precursors and SQ products were completely described. The derived SQs were additionally characterized in thin-film configuration using cyclic voltammetry and UV-VIS spectroscopy and then processed to prepare self-standing bulk heterojunction (BHJ) thin films in conjunction with fullerene-based electron acceptors, which were characterized via profilometry. The comparison between SQ and BHJ solutions and thin films, using atomic force microscopy and UV-VIS spectroscopy, revealed differences in susceptibility for the organization and orientation of the constituting domains.

## 1. Introduction

Solar cells are regarded as an important technology for solving environmental problems and utilizing renewable energy, so various types of solar cells have been developed. Over the last few decades, organic photovoltaics (OPV) have been heavily researched as an alternative to silicon photovoltaics, and significant progress in terms of power conversion efficiency (PCE) and stability has been made [1]. Recently, for single-junction perovskite solar cells, very promising power conversion efficiencies (PCEs) of 25.5% were reported (which is close to the Shockley–Queisser limit), but the stabilities of the silicon devices were not surpassed [2].

The comparative advantages of OPV are mainly their low price, flexibility, transparency, and bendability/stretchability [3,4]. Moreover, applications as an integrated power source for sensors in internet of things applications (IoT) are suggested [5]. To obtain good performance with the OPVs, the electron donor absorbers are used in a bulk heterojunction (BHJ) configuration (maximizing the specific surface of the interface) with fullerene-based electron donors [6]. One of the most promising ways to boost the PCE of the OPV was found through the incorporation of non-fullerene and small molecule donors and acceptors, which exhibit high absorption spectra, resulting in high photocurrents [7,8], with PCEs above 18% in state-of-the-art solar cells [9,10,11], bringing them closer to the market. The most important factors affecting OPV performance are traps and recombination, charge carrier mobility, charge carrier density, and parasitic resistances. Therefore, a few essential steps in the design of the improved SC devices are: the determination of appropriate processing parameters of the constituent layers (experimental conditions, structure modification parameters, and geometric shape optimization), analyses of their contribution to the device performance for further optimization (manufacturing process optimization) [12,13], and improving the nanomorphology of the BHJ layer by inducing increased molecular packing to maximize charge transfer and reduce recombination [14].

In this work, we follow a structure modification strategy. We developed new small molecule photoabsorbers that are based on squaraines (SQ). Even though squaraines have been previously applied as xerographic photoreceptors [15], an optical recording medium [16], electro-light-emitting diodes [17], and nonlinear optical devices [18] on behalf of their unique electronic structures and optical properties, their application in a group of organic photovoltaic devices (OPV) and related devices is still scarce in the literature [19,20]. SQ’s advantages, when considering applications as donor materials in OPVs, are high absorption coefficients, where bandwidths can be adjusted in the visible and NIR range, as well as high photochemical and thermal stability [21]. The seemingly straightforward synthetic pathways are also a nominal advantage. Namely, a variety of SQs can be prepared through the modification of the SQ structure. The replacing of the side groups allows some adjusting of the chemo-physical behavior and opto-electrical properties [22,23], as well as the morphological and organizational properties of the SQs when processed into thin films, which can be used as a valuable tool to optimize the photovoltaic properties of squaraine-based bulk heterojunction absorber layers. Thus, the main tasks of new SQ developments are to: (1) prepare SQs that individually show favorable properties, and (2) ensure that these SQs are compatible with target co-absorbers (acceptors), both in terms of chemical compatibility and in susceptibility for microstructural organization (such as J-aggregate and H-aggregate stacking). Generally, two identical groups can be attached to the squaric acid core to yield symmetric squaraines, or asymmetric squaraines in the case of different groups. It can be said that the BHJ configuration is most frequently used for OPV systems, as the donor/acceptor phase separation in the active layer can easily be optimized to match the diffusion lengths of charge carriers created upon exciton splitting [24]. For blending with donor photoabsorbers, fullerene-based acceptors were most frequently reported, among them the PC_60_BM and PC_71_BM compounds being the mainstream choice due to their high electron affinity, good matching of the HOMO/LUMO levels with squaraine-based materials, and their ability to cover the blue and green region of the absorption spectra (unlike squaraine-based materials), providing an overall much better utilization of the solar spectrum [23]. The difference between PC_60_BM and PC_71_BM mainly lies in the fact that PC_71_BM is more easily processed in solvents and has better absorption in the visible region [25]. The donor/acceptor ratio in the BHJ has to be balanced in order to achieve efficient charge carrier extraction, as SQ derivatives have size-dependent absorption and hole mobility where aggregates and dimers show the poorest functional properties [26].

**Figure 1:**
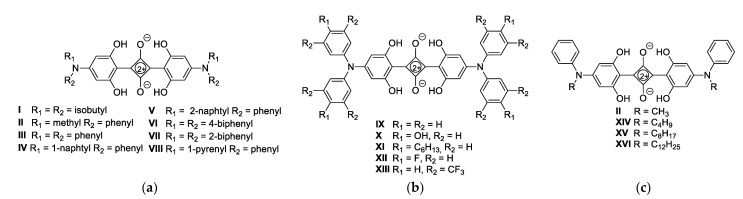
Structures of the previously investigated squaraines: alkyl and aryl anilinosquaraines **I**–**VIII** (**a**); diarylsquaraines **IX**–**XIII** (**b**); and alkylarylsquaraines **II** and **XIV**–**XVI** (**c**).

In recent years, a series of symmetrical and unsymmetrical squaraines have been designed for photovoltaic applications [27]. M. E. Thompson reported the synthesis of *N*,*N*-diaryl- and *N,N*-dialkylanilino squaraines (Figure 1a) [28]. Symmetric squaraine dyes with *N*,*N*-diarylanilino substituents showed optical bandgaps of approximately 2 eV, high solubility, *λ_max_* = 650–750 nm, and high absorptivity in the red solar spectral region, making them promising candidates for application in OPV devices. Asymmetrical *N*,*N*-diisobutylanilino- and *N*,*N*-diphenylanilino-(diphenylamino)squaraines give blueshifted absorption spectra relative to their symmetric counterparts. Lahti and Thayumanavan prepared a series of squaraine dyes with electron-donating (OH, C_6_H_13_) and electron-withdrawing (F, CF_3_) groups, where the absorbance of the dyes (*λ_max_* = 650–700 nm) and the band gap depended significantly on their electron-donating character (Figure 1b) [29]. As the electron-donating character of the dyes increases, the absorbance of the dyes redshifts and the optical band gap decreases from 1.7 eV to 1.4 eV. Furthermore, H. Sasabe and J. Kido reported a synthesis of alkyl-substituted aryl aminosquaraine (*E_gOPTICAL_* = 1.7 eV, *λ_max_* = 650 nm) (Figure 1c) in order to increase the power conversion efficiency from BHJ photovoltaic cells, and they came to the conclusion that the shorter alkyl chains induce a strong redshifting and the broadening of the peak responsible for the enhanced photoresponse in the NIR region [30].

Here, we present the results of the development of new small molecule absorbers from the group of SQs that might represent an answer to the demands for PCE upgrades in the area of squaraine-based OPV devices. We vary the groups attached to the SQ core in the course of the synthesis, which has been specifically developed to ensure the derived products are chemically and microstructurally compatible with other constituents and absorbers in an OPV device. All investigated squaraine dyes, synthesized mostly through three steps (through palladium-catalyzed Buchwald or Ullmann coupling, followed by demethylation with BBr_3_ and condensation with squaric acid) have been fully described. The idea of this paper was to develop a synthesis route for new SQs, avoiding expensive catalytic processes. Therefore, special emphasis was placed on optimizing the amination reaction and developing a simple and mild synthetic route for the preparation of tertiary aryl amines, which were used as starting precursors in further syntheses. Additionally, the SQ and BHJ solutions and thin films have been compared to shed more light on the course of the organization and orientation of the constituting domains, which is critical for the PCE of OPVs. This research will lay a good foundation for further cost-effective synthesis strategies and provide an overview of the preliminary parameters that characterize a good small molecule donor. Furthermore, future structural studies on novel squaraine compounds in model BHJ systems for PV applications are necessary as they contribute to a broader understanding of morphology and nanostructure of SQ-based BHJ layers, paving the way for the incorporation of SQ derivatives into non-model systems, such as non-fullerene small molecule acceptors or even interfacing these with nanostructured charge carrier transport layers.

## 2. Materials and Methods

### 2.1. Preparing

The following chemicals were used: 1-bromo-3,5-dimethoxybenzene 97% (BrC_6_H_3_(OCH_3_)_2_, Acros Organics, Geel, Belgium), *n*-butyllithium 2.5 M solution in hexane (CH_3_(CH_2_)_3_Li, Sigma-Aldrich, St. Louis, MO, USA), 4-benzylpiperidine 99% (C_12_H_17_N, Merck, Darmstadt, Germany), *N*-isopropylbenzylamine (C_6_H_5_CH_2_NHCH(CH_3_)_2_, Sigma-Aldrich, St. Louis, MO, USA), dihexylamine (CH_3_(CH_2_)_5_NH(CH_2_)_5_CH_3_, Acros Organics, Geel, Belgium), boron tribromide 1.0 M solution in methylene chloride (BBr_3_, Sigma-Aldrich, St. Louis, MO, USA), 3,4-dihydroxy-3-cyclobutene-1,2-dione 99.0% ((HO)_2_C_4_(=O)_2_, Acros Organics, Geel, Belgium), chloroform p.a. (CHCl_3_, Sigma Aldrich, St. Louis, MO, USA), acetonitrile anhydrous p.a. (C_2_H_3_N, Sigma Aldrich, St. Louis, MO, USA), 1,2-dichlorbenzene p.a. (C_6_H_4_Cl_2_, Sigma Aldrich, St. Louis, MO, USA), tetrabutylammonium hexafluorophosphate p.a. (C_16_H_36_F_6_NP, Sigma Aldrich, St. Louis, MO, USA), ferrocene 98% (Fe (C_5_H_5_)_2_, Sigma Aldrich, St. Louis, MO, USA), [6,6]-phenyl-C_71_-butyric acid methyl ester (C_82_H_14_O_2_, Ossila, Sheffield, UK), and indium tin oxide (ITO)-coated glass substrates (15 Ohm/sq, Lumtec, New Taipei City, Taiwan).

#### 2.1.1. Synthesis of Compounds **1a**–**1c**

**Figure 2:**
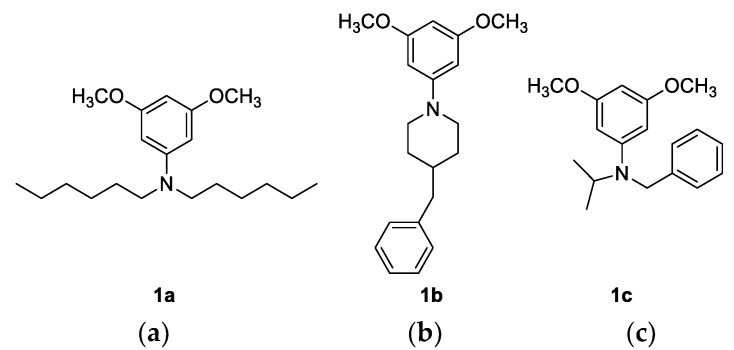
Structures of the derived products: (**a**) *N*,*N*-dihexyl-3,5-dimethoxyaniline (**1a**): 45%; ^1^H NMR (CDCl_3_; 600 MHz) *δ*/ppm: 6.85 (s, 3H, H-ar), 3.78 (s, 6H, –OCH_3_), 3.22 (t, *J* = 7.6 Hz, 4H), 1.56–1.62 (m, 4H), 1.29–1.35 (m, 12H), 0.9 (t, *J* = 6.7 Hz, 6H); ^13^C NMR (CDCl_3_, 150 MHz) *δ*/ppm: 161.22 (s), 149.2 (s), 90.64 (d), 86.68 (d), 54.59 (q), 50.72 (t), 31.25 (t), 26.82 (t), 26.37 (t), 22.20 (t), 13.55 (q); HRMS: M^+^_calcd_ 322.2741; M^+^_found_ 322.2741; (**b**) 4-benzyl-1-(3,5-dimethoxyphenyl)piperidine (**1b**): 55%; ^1^H NMR (CDCl_3_; 600 MHz) *δ*/ppm: 7.29 (td, *J* = 7.5 Hz; 1.4 Hz, 2H, H-ar), 7.20 (td, *J* = 7.5 Hz; 1.4 Hz, 1H, H-ar), 7.16 (dd, *J* = 7.5 Hz; 1.4 Hz, 2H, H-ar), 6.09 (d, *J* = 2.1 Hz, 2H, H-ar), 5.96 (t, *J* = 2.1 Hz, 1H, H-ar), 3.76 (s, 6H, –OCH_3_), 3.61–3.66 (m, 2H), 2.64 (td, *J* = 12.7 Hz; 2.6 Hz, 2H), 2.57 (d, *J* = 7.0 Hz, 2H), 1.70–1.75 (m, 2H), 1.35–1.43 (m, 2H); ^13^C NMR (CDCl_3_, 150 MHz) *δ*/ppm: 161.42 (s), 153.71 (s), 140.45 (s), 129.15 (d), 128.23 (d), 125.90 (d), 95.41 (d), 91.17 (d), 55.21 (q), 49.90 (t), 43.16 (t), 38.00 (d), 31.93 (t); HRMS: M^+^_calcd_ 312.1958; M^+^_found_ 312.1959; (**c**) *N*-benzyl-*N*-isopropyl-3,5-dimethoxyaniline (**1c**): 80%; ^1^H NMR (CDCl_3_; 300 MHz) *δ*/ppm: 7.15–7.32 (m, 5H, H-ar), 6.89 (d, *J* = 2.0 Hz, 2H, H-ar), 5.87 (t, *J* = 2.0 Hz, 1H, H-ar), 4.39 (s, 2H), 4.16–4.27 (m, 1H), 3.68 (s, 6H), 1.2 (d, *J* = 6.7 Hz, 6H); ^13^C NMR (CDCl_3_, 150 MHz) *δ*/ppm: 157.30, 151.25, 149.75, 128.41, 126.39, 126.17, 92.62, 88.50, 56.12, 55.08, 48.55, 48.46, 20.00; HRMS: M^+^_calcd_ 286.1802; M^+^_found_ 286.1809.

Here, 1.0 g (0.0047 mol) of 1-bromo-3,5-dimethoxybenzene was dissolved in 30 mL of dry diethyl ether, followed by the addition of the corresponding amine (1.5 eq), after which n-BuLi (1.5 eq, 2.5 M solution in hexane) was added dropwise in a stream of nitrogen at 0° C. After 2 h of stirring, 10 mL of water were added to remove unreacted n-BuLi. The reaction mixture was extracted with diethyl ether (2 × 10 mL) and water (2 × 10 mL) and dried over MgSO_4_. After the evaporation of the solvent, the resulting products **1a**–**1c** were purified by column chromatography on silica gel with petroleum ether as the eluent (Figure 2).

#### 2.1.2. Synthesis of Compounds **2a**, **2b**, **3b**, and **3c**

**Figure 3:**
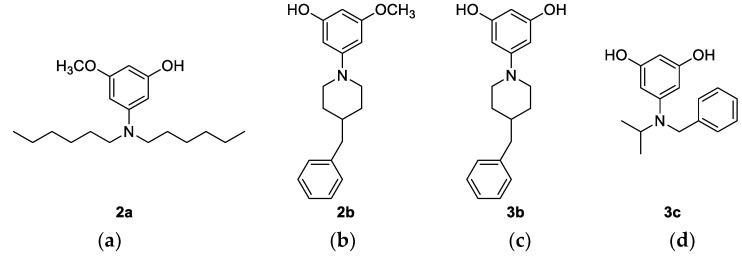
Structures of the derived products: (**a**) 3-(dihexylamino)-5-methoxyphenol (**2a**): 20%; ^1^H NMR (CDCl_3_; 600 MHz) *δ*/ppm: 7.07 (s, 1H, H-ar), 6.61 (s, 1H, H-ar), 6.54 (s, 1H, H-ar), 3.81 (s, 3H, –OCH_3_), 3.40–3.50 (m, 2H), 3.11–3.24 (m, 2H), 3.11–3.24 (m, 4H), 1.16–1.38 (m, 18H); ^13^C NMR (CDCl_3_, 150 MHz) *δ*/ppm: 162.06, 159.53, 103.02, 102.51, 99.40, 59.20, 55.85, 31.07, 26.19, 24.66, 22.42, 13.87; HRMS: M^+^_calcd_ 308.2584; M^+^_found_ 308.2583; (**b**) 3-(4-benzylpiperidin-1-yl)-5-methoxyphenol (**2b**): 55%; ^1^H NMR ((CD_3_)_2_CO; 600 MHz) *δ*/ppm: 7.10–7.30 (m, 5H, H-ar), 7.16 (s, 2H), 6.55 (s, 1H), 3.80 (s, 3H, –OCH_3_), 3.52–3.62 (m, 4H), 2.62 (d, *J* = 7.12 Hz, 2H), 2.17–2.34 (m, 4H), 1.88 (d, J = 13.4 Hz, 2H), 2.82–3.34 (m, 3H); ^13^C NMR ((CD_3_)_2_CO, 150 MHz) *δ*/ppm: 162.58 (s), 160.66 (s), 145.54 (s), 140.68 (s), 130.00 (d), 129.29 (d), 127.03 (d), 103.23 (d), 102.24 (d), 99.90 (d), 62.16 (t), 57.17 (t), 56.35 (q), 35.69 (t), 19.76 (t), 14.31 (t); HRMS: M^+^_calcd_ 298.1802; M^+^_found_ 298.1802; (**c**) 5-(4-benzylpiperidin-1-yl)benzene-1,3-diol (**3b**): 45%; ^1^H NMR ((CD_3_)_2_CO; 600 MHz) *δ*/ppm: 7.10–7.30 (m, 5H, H-ar), 6,96 (s, 2H), 6.46 (s, 1H), 3.52–3.62 (m, 4H.), 2.62 (d, *J* = 7.12 Hz, 2H), 2.17–2.34 (m, 4H.), 1.88 (d, *J* = 13.4 Hz, 2H), 2.82–3.34 (m, 3H); ^13^C NMR ((CD_3_)_2_CO, 150 MHz) *δ*/ppm: 160.47 (s), 145.67 (s), 140.76 (s), 129.94 (d), 129.22 (d), 126.96 (d), 104.50 (d), 101.00 (d), 62.11 (t), 57.00 (t), 35.88 (t), 19.71 (t), 14.21 (t); HRMS: M^+^_calcd_ 284.1645; M^+^_found_ 284.1646; (**d**) 5-(benzyl(isopropyl)amino)benzene-1,3-diol (**3c**): 60%; ^1^H NMR ((CD_3_)_2_CO; 300 MHz) *δ*/ppm: 7.67–7.76 (m, 3H, H-ar), 7.15–7.28 (m, 5H, H-ar), 4.10–4.21 (m, 1H), 3.73 (d, *J* = 2.80 Hz, 2H), 3.31 (s, 6H); ^13^C NMR (CD_3_OD, 150 MHz) *δ*/ppm: 159.59 (2C), 137.94, 130.90 (2C), 129.73, 129.31 (2C), 128.32 (2C), 62.58, 58.69, 17.72, 17.65; HRMS: M^+^_calcd_ 258.1489; M^+^_found_ 258.1488.

To a solution of corresponding amine **1a**–**1c** in dry dichloromethane (0.75 M), BBr_3_ was added dropwise in a stream of nitrogen at −78 °C, and the mixture was stirred for 3 h, gradually warming to room temperature. The reaction was quenched by methanol (1 mL) and concentrated under vacuum. The obtained alcohols (**2a**, **2b**, **3b**, and **3c**) were separated *via* column chromatography on silica gel with dichloromethane as the eluent, and they were used without further purification (Figure 3).

#### 2.1.3. Synthesis of Compounds **4a**, **4b**, **5b**, and **5c**

**Figure 4:**
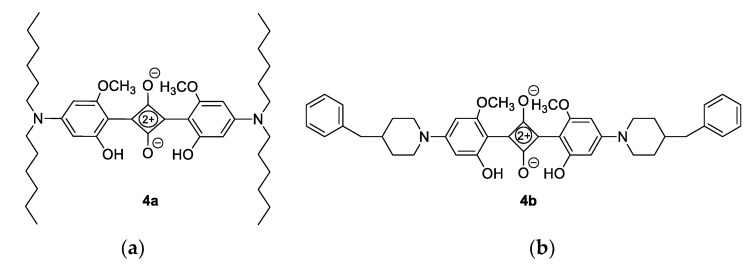

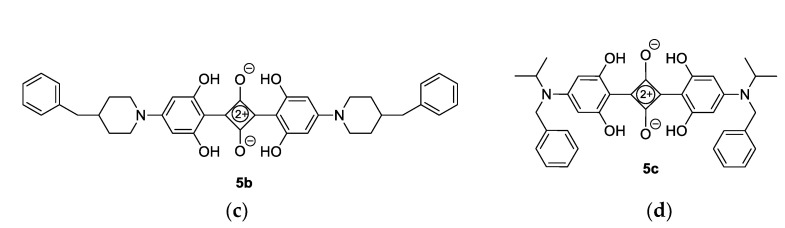
Structures of the derived products: (**a**) 2,4-bis(4-(dihexylamino)-2-hydroxy-6-methoxyphenyl)cyclobuta-1,3-diene-1,3-bis(olate) (**4a**): 10%; ^1^H NMR (CDCl_3_; 600 MHz) *δ*/ppm: 5.78 (d, 2H, *J* = 1.9, H-ar), 5.59 (d, 2H, *J* = 1.9, H-ar), 3.90 (s, 6H, OCH_3_), 3.30–3.37 (m, 8H), 1.60–1.67 (m, 8H), 1.29–1.36 (m, 24H), 0.86–0.94 (m, 12H); ^13^C NMR (CDCl_3_, 150 MHz) *δ*/ppm: 156.57 (s), 93.89 (d), 87.63 (d), 55.48 (q), 51.50 (t), 31.55 (t), 27.82 (t), 26.69 (t), 22.59 (t), 13.99 (q); HRMS: M^+^_calcd_ 692.4775; M^+^_found_ (+1H) 693.4848; (**b**) 2,4-bis(4-(4-benzylpiperidin-1-yl)-2-hydroxy-6-methoxyphenyl)cyclobuta-1,3-diene-1,3-bis(olate) (**4b**): 20%; ^1^H NMR (CDCl_3_; 300 MHz) *δ*/ppm: 7.09–7.39 (m, 10H, H-ar), 5.93 (s, 2H, H-ar), 5.73 (s, 2H, H-ar), 3.99 (d, *J* = 13.0 Hz, 4H), 2.96 (t, *J* = 13.0 Hz, 4H), 2,57 (d, *J* = 6.9 Hz, 4H), 1.72–1.93 (m, 10H); ^13^C NMR (CD_3_OD, 150 MHz) *δ*/ppm: 162.06, 162.02, 159.91, 159.87, 139.14, 128.83 (2C), 128.09 (2C), 125.97, 99.94, 97.74, 55.87, 54.80, 41.52, 34.85, 29.55; HRMS: M^+^_calcd_ 672.3210; M^+^_found_ (+1H) 673.3253; (**c**) 2,4-bis(4-(4-benzylpiperidin-1-yl)-2,6-dihydroxyphenyl)cyclobuta-1,3-diene-1,3-bis(olate) (**5b**): 20%; ^1^H NMR ((CD_3_)_2_SO; 600 MHz) *δ*/ppm: 7.00–7.40 (m, 14H, H-ar), 5.75 (s, 4H, H-ar), 3.40–3.60 (m, 4H), 1.50–1.66 (m, 8H), 1.17–1.32 (m, 4H); ^13^C NMR (CD_3_OD, 150 MHz) *δ*/ppm: 205.97, 191.27, 190.74, 72.00, 31.84, 18.25, 12.60 (due to the poor solubility of the compound, all signals could not be completely detected); HRMS: M^+^_calcd_ 644.2897; M^+^_found_ (+1H) 645.2935; (**d**) 2,4-bis(4-(benzyl(isopropyl)amino)-2,6-dihydroxyphenyl)cyclobuta-1,3-diene-1,3-bis(olate) (**5c**): 20%; ^1^H NMR (CDCl_3_; 300 MHz) *δ*/ppm: 10.92 (s, 4H, –OH), 7.10–7.37 (m, 10H, H-ar), 4.87 (s, 4H, H-ar), 4.58 (s, 4H), 4.31–4.43 (m, 2H), 3.60–3.71 (m, 12H); ^13^C NMR (CDCl_3_, 150 MHz) *δ*/ppm: 190.16, 185.04, 184.50, 129.65, 129.47, 128.81 (2C), 126.87 (2C), 74.68, 32.47, 19.18 (2C); HRMS: M^+^_calcd_ 592.2579; M^+^_found_ (+1H) 593.2651.

To a solution of the corresponding alcohol **2a**, **2b**, **3b**, and **3c** in a toluene/butanol mixture (13.00 M), squaric acid (0.5 eq) was added, and the mixture was stirred for 16 h at a reflux temperature. After cooling to room temperature and the evaporation of the solvent, the resulting products **4a**, **4b**, **5b**, and **5c** were purified via column chromatography on silica gel with petroleum ether as the eluent (Figure 4).

### 2.2. Deposition

The synthesized SQs were dissolved together with PC_71_BM at a weight ratio of 1:6 in either chloroform or o-DCB (1,2-dichlorbenzene) in the total concentrations shown in Table 1. Upon intense stirring for 48 h, the solutions were filtered and spin cast in a protective atmosphere (N_2_) onto ITO glass substrates that were previously ultrasonically cleaned in isopropyl alcohol and etched with oxygen plasma. Different solvents and spin-coating parameters shown in Table 1 were chosen in order to gain insight into the ability of different solvents to produce thin films at a wide range of thickness, from 50 to 200 nm, as the optimal film thickness for the absorbing layer in an OPV generally falls into this range.

### 2.3. Characterization

The ^1^H NMR spectra were recorded on the spectrometers Bruker AV600 and AV300 (Mannheim, Germany). The ^13^C NMR spectra were registered at 75 and 150 MHz. All NMR spectra were measured in CDCl_3_ using tetramethylsilane as a reference.

Silica gel (0.063–0.2 mm) was used for chromatographic purifications. Thin-layer chromatography (TLC) was performed using silica gel 60 F254 plates. Solvents were purified through distillation.

The samples’ exact masses were measured on the Agilent (Santa Clara, CA, USA) 6545 Q-TOF LC/MS (G6545B) connected to an Agilent 1290 Infinity II UPLC system (high-pressure pump G7120A, autosampler G7129B, column compartment G7116B, and DAD detector G7117B). All samples were dissolved in pure methanol. Elution was performed on a Phenomenex Luna Phenyl-Hexyl chromatographic column (250 mm × 4.6 mm, 5 μm), using the following mobile phases: 0.1% acetic acid in water as mobile phase A and 100% methanol as mobile phase B. The gradients used at the flow of 1 mL min^−1^ for the separation of compounds on the column are shown in Table 2.

The exact masses of the eluted compounds were recorded on an Agilent (Santa Clara, CA, USA) 6545 Q-TOF detector using the following parameters of the method: the gas temperature of the source was set to 300 °C, 8 L min^−1^ was the flow of the drying gas, the nebulizer pressure was set to 60 psi, and the sheath gas temperature was set to 300 °C and 7 L min^−1^ of flow. The capillary voltage was set to 3000 V and the nozzle voltage was set to 1000 V. The fragmentor was set at 100 and 80 V, and the skimmer was set to 65 V. The fragmentor setting was affecting the recovery of ions of interest, which is why the samples were measured at two different values. The masses were acquired between 40 and 1700 *m*/*z* at an acquisition rate of 1.5 spectra/min and 666.7 ms/spectrum. The reference mass correction was performed using a tuning solution with an *m*/*z* of 121.050873 and 922.009798 to cover the range of masses of all the samples. The reference masses were within 5 ppm of error in all the recorded chromatograms.

To record absorption/excitation and emission spectra, samples were measured on an Agilent (Santa Clara, CA, USA) 1290 Infinity I UPLC system (high-pressure pump G4220A, autosampler G4226A, column compartment G1316C, DAD detector G7117A, and FLD detector G1321B). The same column and chromatographic elution conditions were used as for the MS measurements. To record fluorescent spectra, 360 and 450 nm excitation wavelengths were used to record emission and excitation spectra, respectively. These wavelengths were chosen during method development as the optimal wavelength for the compounds of interest.

Surface profilometry measurements were performed on a DektakXT stylus system for profiling (Bruker, Billerica, MA, USA) equipped with a 12.5 μm-radius stylus tip to determine the layer thickness and surface roughness of thin film samples. Line scans were recorded over a length of 2000 μm, with a stylus force of 3 mg and a resolution of 0.33 μm per pt. Layer thickness and surface roughness values were derived from surface profiles. For surface profilometry, the thin films were blends of squaraine and PC_71_BM dissolved in either chloroform or *o*-DCB spin cast at varying conditions.

The absorption spectra of thin films were recorded on an UV-1800 UV-VIS spectrophotometer (Shimadzu, Kyoto, Japan) in the range of 450–1000 nm. The films were prepared by drop casting 5 mg/mL pristine squaraine solutions followed by evaporation. 

Cyclic voltammetry was measured on a SP-50 potentiostat/galvanostat (BioLogic Seyssinet-Pariset, France) in anhydrous acetonitrile with tetrabutylammonium hexafluorophosphate (Bu_4_NPF_6_, 0.1 M) as a supporting electrolyte. The setup consisted of three electrodes: a platinum wire counter electrode, an Ag/Ag^+^ (0.01 M AgNO_3_ in anhydrous acetonitrile) reference electrode, and drop-cast films of squaraine materials in chloroform (5 mg mL^−1^) on a platinum surface as the work electrode. The reference electrode was calibrated against a ferrocene/ferrocenium solution (Fc/Fc^+^) in the aforementioned solution. The measurements were performed in an inert atmosphere (N_2_) in the range from −2.5 to 2.5 V at a scan rate of 50 mV s^−1^. The HOMO/LUMO levels were estimated from the onset of the oxidation and reduction potential (*E*_ox_ and *E_red_*) of the squaraine thin films, considering the energy level of Fc/Fc^+^ to be −4.8 eV below the vacuum level with the equations listed below. LUMO levels were also calculated as the difference between the optical bandgap determined via UV/Vis and the HOMO level determined via cyclic voltammetry.

(1) $$E_{HOMO}=-\left[\left(E_{ox}-E_{\frac{1}{2}ox\left(Fc\right)}+4.8\right)\right]\mathrm{eV}$$

(2) $$E_{LUMO}=-\left[\left(E_{red}-E_{\frac{1}{2}ox\left(Fc\right)}+4.8\right)\right]\mathrm{eV}$$

Atomic force microscopy (AFM) measurements were performed on a Tosca™ 400 atomic force microscope (Anton Paar, Graz, Austria) in tapping mode using Al-coated cantilevers (ARROW-NCR, NanoWorld AG, Neuchâtel, Switzerland) with a resonance frequency of 285 kHz and a force constant of 42 N m^−1^. All measurements were acquired at room temperature under ambient conditions. All calculations and image processing were performed with Tosca™ analysis software V7.4.8341 (Anton Paar, Graz, Austria).

## 3. Results

### 3.1. Developing the Synthesis

#### 3.1.1. Amine Preparation

**Figure 5:**
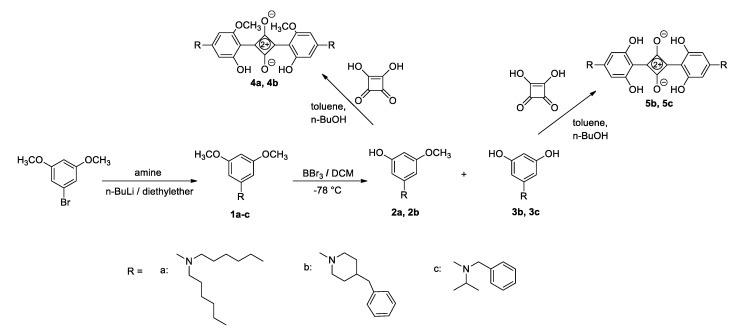
Synthesis of squaraines **4a**, **4b**, **5b** and **5c**.

The synthetic procedures for squaraines **4** and **5** included several steps, starting with the preparation of the amino precursors **1a**–**1c** (Figure 2). Traditionally, the most used methods for the preparation of tertiary aryl amines mainly included a transition metal-catalyzed amination reaction, usually requiring high temperatures and long reaction times. Buchwald–Hartwig amination utilizing a palladium catalyst was literarily the most used method for the synthesis of amine precursors in squaraine chemistry. The major drawback of the Buchwald–Hartwig reaction is the large amount of catalyst required and their separation from organic products, which is of particular importance because of their residual toxicity in the target compounds. Moreover, transition metal-catalyzed reactions also generate hazardous waste, which is environmentally problematic and should be avoided wherever possible. To avoid the use of catalysts and rigid reaction conditions, we investigated the preparation of the amines with an alternative milder and cheaper procedure. After a series of experimental attempts (changes in reaction conditions, solvents, amines, and attempt at microwave synthesis) with limited success, the amination reaction has been successfully conducted using an optimized method based on the Pittelkow method [27]. Unlike previously performed experiments, in this case, the catalysts were not used, and the reaction was carried out in one step between halogen and corresponding amine with the addition of n-BuLi [31]. On behalf of this mild procedure, for which the transition metal catalyst was not necessary, amines **1a**–**1c** have been successfully prepared with a very good yield (Figure 5).

**Figure 6:**
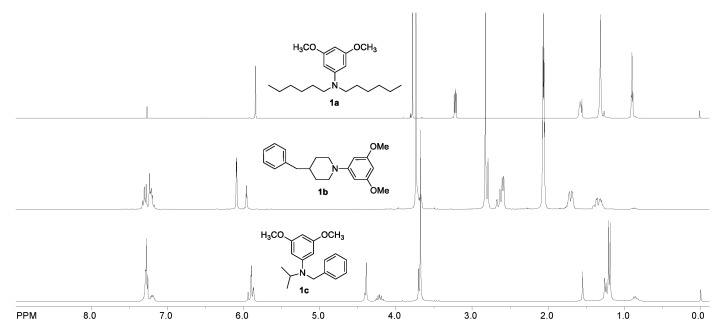
^1^H NMR spectra (CDCl_3_) of compounds **1a**–**1c**.

Figure 6 presents the ^1^H NMR spectra of compounds **1a**–**1c**. The influence of substituents on crucial proton signals was not significant. In all cases, characteristic aromatic protons appear at about 6 ppm, while the methoxy group can be observed at about 4 ppm. Other signals refer to the proton substituents on nitrogen.

#### 3.1.2. Squaraine Preparation

Since previous research has shown that hydroxy derivatives are more thermally stable precursors than methoxy derivatives, the next step of the synthesis concerned the demethylation process. The demethylation of substrates **1a**–**1c** was performed according to a conventional procedure using BBr_3_ in dichloromethane, wherein the degree of demethylation and the number of formed hydroxyl groups depended on the structure of the starting compound. Therefore, in the case of the dihexyl derivative **1a**, only the mono-hydroxyl compound **2a** was obtained. The piperidine derivative **1b** gave a mixture of mono- and di-demethylated products **2b** and **3b**, in contrast to the demethylation reaction of compound **1c**, where only the di-demethylated product **3c** was isolated (Figure 3). All four hydroxyl derivatives **2a**, **2b**, **3b**, and **3c** were used without further purification in the final step of the squaraine synthesis. The condensation reaction of squaric acid and the corresponding amines **2a**, **2b**, **3b**, and **3c** (Figure 3) has been conducted according to the standard procedure in a toluene/butanol mixture, whereby the target squaraines **4a**, **4b**, **5b**, and **5c** were formed as the main products (Figure 4). The products were further purified via column chromatography and completely characterized using spectroscopic methods.

#### 3.1.3. UV-VIS Spectroscopic Characterization of the SQ Dyes

**Figure 7:**
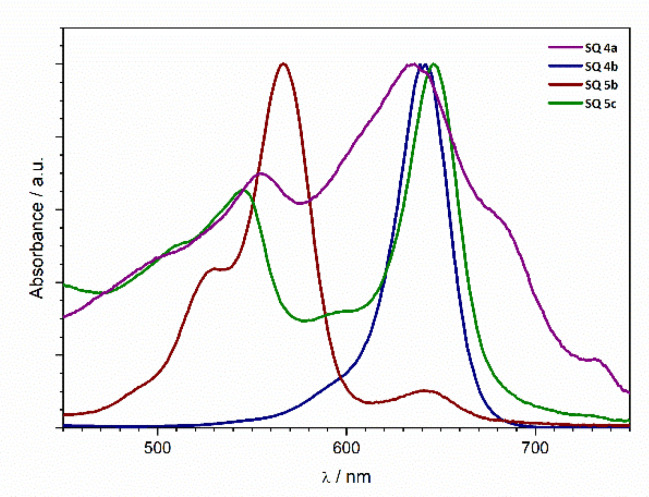
Normalized absorption spectra of compounds **1a**–**1c** (in ethyl alcohol).

The UV-VIS spectra of the prepared squaraines **4a**, **4b**, **5b**, and **5c** show absorption maxima in the characteristic area from 500 to 700 nm (Figure 7), which makes them excellent candidates for further characterization as photovoltaic materials. Squaraine **4b** shows a significantly blueshifted absorption maximum in ethanol relative to the products **4a**, **5b**, and **5c**. However, a noticeable local maximum of absorption can be observed in the 650–700 nm range, matching the absorption maxima of other products. 

**Figure 8:**
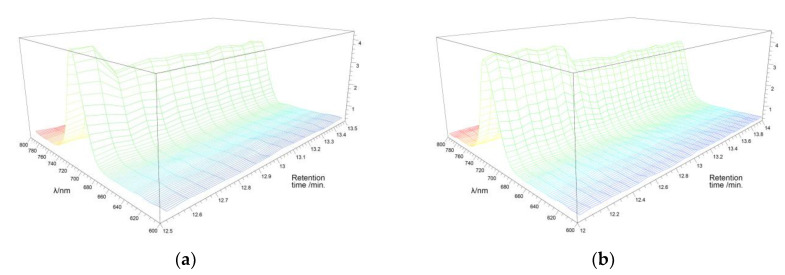
Three-dimensional emission spectra of: squaraines **4b** (**a**) and **5b** (**b**).

Figure 8 presents the 3D emission spectra of products **4b** and **5b** as representative examples to show that no significant influence of substituents and number of hydroxyl groups on emission was observed, furthermore demonstrating that the aniline attached to the squaric core is primarily responsible for such high absorption of synthesized compounds. All spectra show a characteristic maximum at 720 nm.

### 3.2. Developing the Films

#### 3.2.1. Solvent Selection, Blending Ratio, and Deposition Conditions

The course of the optimization was performed for the purpose of blending the selected donor SQs with fullerene-based acceptors. A number of reports where the PCBM-to-SQ ratio was investigated suggest that a 1:(x > 5) of SQ:PCBM is mostly favored as the PCBM matrix suppresses SQ aggregation, which consequently increases the overall functional properties of the BHJ, regardless of the lesser amount of donor material in the active layer [23,24,25,26]. For the purposes of developing a method for spin coating these blends, the ratio of 1:6 was used. Since compounds **4a**, **5b**, and **5c** are quite similar in terms of the interaction with the acceptor material and solvent, we find that the film thickness and surface roughness are mainly functions of the solvent, PC_71_BM concentration, process conditions, and amount of, but not the type of, SQ material. A notable difference in the solubility of compound **4b** in chloroform and chlorobenzene was observed through spin casting, as the thin films cast from an apparently homogeneous solution produced a granular morphology with visibly noticeable crystals of sizes up to a few hundred nanometers, indicating that no primary nucleation took place during film formation. Instead, the undissolved small aggregate particles served as primary nuclei, which resulted in much bigger SQ domains. This is also supported by the significant blueshift of the SQ **4b** absorption maxima, where it can clearly be seen that this compound exhibits different interactions with the solvent used in comparison to squaraines **4a**, **5b**, and **5c**. Apparent homogeneity was held responsible for the beneficial appearance of the films. Namely, the PCE and fill factor of solar cells greatly depend on the homogeneity and domain size of the constituents in the BHJ photoabsorber layer. Having better absorber homogeneity is a good indicator that thicker films can be used without considerably increasing susceptibility for recombination, thus increasing the photocurrent. What can be seen from the preliminary results of profilometry is that, as expected, film thickness and surface roughness decrease with the increase in acceleration and rotation speed during spin coating. As can be seen in Table 1, chloroform as a solvent shows the potential to allow the preparation of thin films in the desired thickness range for BHJ OPV devices (at least 50 nm); however, the drawback is the increased surface roughness of the films in the higher range of concentrations, which introduces a number of practical problems in terms of device fabrication. The BHJ thin films spin coated using *o*-DCB as solvent also showed satisfactory surface roughness (<10 nm), but they exhibited thicknesses much lower than those spin coated using chloroform as a solvent, where the achieved thicknesses definitely fall below the lower range of desired thickness. For that reason, further optimization was desired with the *o*-DCB solvent used for squaraines **4a**, **5b**, and **5c**. The root-mean-square roughness of the films was, according to profilometry measurements, below 10 nm (Table 1) for all the films spin coated from solutions of 20 mg mL^−1^ concentration, while the values go up by approximately an order of magnitude higher (50–100 nm) when the thin films were spin coated from solutions of double the concentration (40 mg mL^−1^). In the case that films thicker than 100 nm are to be optimized, it will be necessary to strike a balance between increasing the concentration and lowering the spin-coating speed and/or acceleration values in order to increase film thickness and minimize surface roughness.

#### 3.2.2. Optical, Electrical, and Surface Characterization of SQ in Thin-Film Configuration

To showcase the potential for application in photovoltaic devices, the most important preliminary parameters (HOMO/LUMO levels and the optical bandgaps of the synthesized compounds) were estimated on drop-cast thin films via UV-VIS spectroscopy and cyclic voltammetry, and the results are shown below. In this work, we display a basic optical characterization, a more detailed investigation of the effects of thickness on the absorption, and optimal BHJ morphology (H-aggregate and J-aggregate stacking), which requires closed SCs and a top-down course of substantial investigation, and which will be reported in a follow-up study. However, we already observed a group of quite unexpected parameters governing the mechanisms behind the successful development of the homogeneous and applicable BHJ absorbers based on SQs, but again, due to the extent of the results, this will be reported separately.

**Figure 9:**
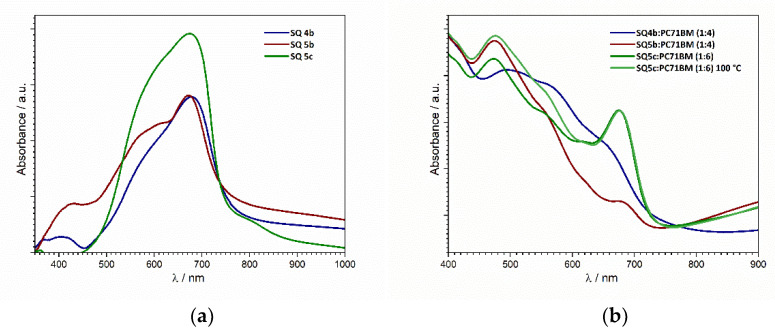
(**a**) Normalized UV-VIS spectra of pristine squaraine drop-cast thin films. The optical bandgap was determined from the onset values (SQ **4a** could not be drop-cast); (**b**) UV-VIS spectra of BHJ thin films in different ratios and post-processing treatments spin-coated from solutions in chloroform.

The UV-VIS spectra of drop-cast thin films in Figure 9a show closely grouped absorption maxima at 672, 675, and 680 nm for compounds **5b**, **5c**, and **4b**, respectively. It can also be seen that the broad absorption peaks extend over a 300 nm range. This is characteristic for squaraine dyes when cast as thin films, while they reveal quite sharp absorption peaks in the bulk or solution [26]. The bandgaps determined from the onset values are shown in Table 3 above and are estimated to be slightly above 1.6 eV for all compounds.

The UV-VIS spectra were additionally utilized for the comparison of contributions between pristine SQ and BHJ films (Figure 9b) to show that different systems show different susceptibilities for the organization and ordering of the constituting domains. By comparing the absorption data of SQ in these different systems/setups, it can be seen that in the BHJ system (Figure 9b), the SQ5c peak (675 nm) is more pronounced and narrow, which indicates less agglomeration (due to its suppression by the PC_71_BM matrix) and more monomeric absorption in comparison to the pristine SQ drop-cast films (Figure 9a). Additionally, to display the existence of the superstructural assembly for compound **5c**, a UV-Vis comparison of BHJ thin films as-cast and BHJ thin films annealed at 100 °C is shown, where a slight bathochromic (red) shift of about 5 nm for the annealed monomer peak indicates oriented stacking (J-aggregates). The agglomeration in all of the BHJ thin films is still present in the form of separate peaks in the wavelength range of 400–600 nm (with less intensity when compared with pristine SQ films due to a lower number of agglomerated particles), but this cannot be seen clearly, as their absorption is interfered with by PC_71_BM absorption. However, from the UV-Vis spectra in ethanol, in which most of the compounds are not completely soluble, the optical absorption of aggregates can be observed, and for all of the compounds, it ranges from 500 to 600 nm. Additionally, absorption spectra suggest that chloroform is not an optimal solvent for all of these compounds, which can also be seen in Figure 9b. Compounds **4b** and **5b** show pronounced aggregate absorption and very low levels of monomer absorption, indicating that the particles may have not been completely dissolved before the deposition, leading to their more heterogeneous distribution throughout the PC_71_BM matrix. Therefore, by pinpointing the contribution of PC_71_BM to the organizational behavior of SQ, it is possible to confirm that the type of compound, BHJ ratio, choice of solvent, type and extent of post processing, etc., surely play a major role in affecting the aforementioned organizational susceptibilities. With the influence of all of these parameters in mind, it is needless to say that a proper description of the superstructural assembly for different systems will require a much broader investigation with more suitable characterization tools. 

**Figure 10:**
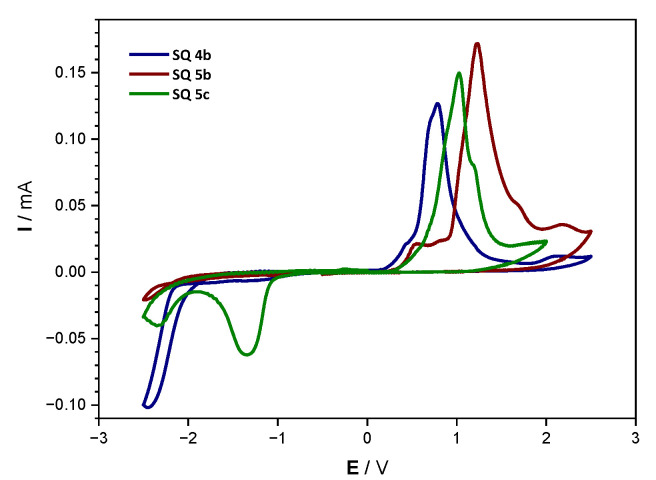
Voltammograms of the drop-cast thin films of pristine squaraine compounds measured using cyclic voltammetry.

Voltammograms obtained via cyclic voltammetry are shown in Figure 10, while the calculated HOMO/LUMO levels and the corresponding bandgaps obtained via this method are shown in Table 3. Due to the fact that the compounds show certain solubility in acetonitrile, the oxidation and reduction peak had to be measured in two separate scans. Additionally, even after separately measuring oxidation and reduction, it was observed that during the reductive scan, compounds **4b** and **5b** completely changed color from blue to red, which implies a reaction or decomposition in a reducing environment. This explains why the LUMO levels for compounds **4b** and **5b** could not be determined directly from the CV measurements. During the oxidative scan, on the other hand, no change in the color of the films were observed, and the results obtained are appropriate for the determination of the HOMO levels of −5.07, −5.19, and −5.34 eV, for compounds **4b**, **5b**, and **5c**, respectively, and these values are comparable to other small molecule squaraine compounds published so far [32,33]. These values are also supported by the fact that compound **5c** displayed complete stability during both the forward and backward scans, and the bandgaps resulting from both methods, the optical and electrochemical, are very similar. Finally, it can be seen that all compounds exhibit relatively deep HOMO levels and low bandgap values, which is beneficiary for photovoltaic application as it allows for better compatibility with the solar spectrum, leading to good absorption and, consequently, charge carrier generation.

**Figure 11:**
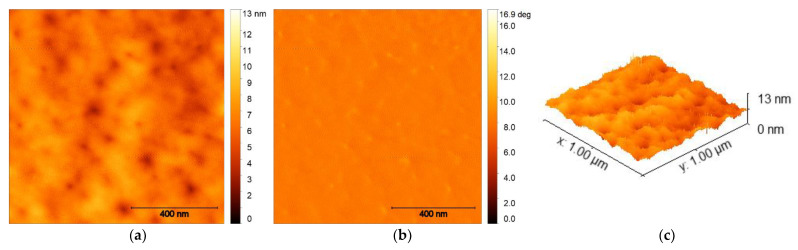
AFM topography of SQ **5c**:PC_71_BM blends that are spin-coated from a 20 mg mL^−1^ solution in chloroform at 2000 rpm: (**a**) height, (**b**) phase, and (**c**) height, 3D view.

AFM characterization was performed on optimized films to shed more light on the surface topography of the spin-coated SQ:PC_71_BM blends. Micrographs show very similar topography for all films spin coated from a 20 mg mL^−1^ chloroform solution, regardless of the BHJ ratio (Figure 11a). Figure 11b,c also shows representative phase scans and 3D height scans for the non-annealed samples. It can be seen that the as-prepared films are very smooth and exhibit very low squared surface roughness which, according to AFM, averages at about 0.77 nm. Furthermore, the RMS of all of the spin-cast films of the same concentration was determined to be <1 nm. This indicates that there is no phase separation, and the blending of the BHJ constituents was confirmed to be successful. Regardless of the miscibility of these blended compounds, morphology and vertical phase separation are of great importance for the enhancement of small molecule donor organic solar cells, and control over these phenomena must be fully achieved to obtain maximum performance; however, here, we confirm that very fine morphology can be reproduced for all compounds, whereas rougher morphology (if necessary) can be achieved through post-process treatment, such as thermal treatment and solvent vapor annealing.

## 4. Conclusions

The main focus of this study was to develop the synthesis of new squaraines, avoiding expensive catalytic processes. Therefore, special emphasis was placed on optimizing the amination reaction and developing a simple and mild synthetic route for the preparation of tertiary aryl amines, which were used as starting precursors in subsequent syntheses. The investigated squaraine dyes (SQs) have typically been synthesized through three steps: through the amination reaction of starting precursors, followed by demethylation with BBr_3_, and then condensation with squaric acid. We varied the groups attached to the SQ core in a course of the synthesis specifically developed to ensure that the derived products can be solution-processed and are chemically and microstructurally compatible with other absorbers and OPV device constituents. All SQ precursors and SQ products were fully described. 

As expected, SQ products generally showed relatively deep HOMO levels and low bandgap values. Bearing in mind their applicability in OPV, SQs were blended with fullerene acceptors, and the optimal ratios, processing solvents, and spin-coating deposition parameters were determined. We indicated the existence of the differences in susceptibility for the organization and orientation of the constituting domains for SQ and BHJ solutions and thin films. Finally, it was evident that there are several strategies available for upgrading the PCE of squaraine-based OPV devices, one of which is developing modular small molecule absorbers that are chemically and microstructurally compatible with the acceptor material in the absorber layer.

## Figures and Tables

**Table 1:** Denomination and parameters of the prepared samples. An approximate thickness indicator calculated on behalf of deposition parameters. Root-mean-square roughness (RMS) is shown for groups of samples in similar ranges.

Sample	Solvent	Total Concentration (mg mL^−1^)	Speed (rpm)/ramp (rpms^−1^)	Film Thickness (nm)	Average RMS/nm
1 (**5c**)	CF	20	1000/1000	110	1–5
2 (**5b**)	CF	20	1000/2000	90	
3 (**5c**)	CF	20	1000/3000	80	
4 (**5c**)	CF	20	3000/3500	50	
5 (**4b**)	CF	20	3000/2000	80	
6 (**4b**)	CF	20	2000/1000	90	
7 (**5b**)	CF	20	2000/2000	70	
8 (**5c**)	CF	40	2000/2000	250	50–100
9 (**5c**)	CF	40	3000/2000	150	
10 (**5c**)	CF	40	1000/3000	200	
11 (**5b**)	*o*-DCB	20	3000/3000	20	5–10
12 (**5b**)	*o*-DCB	20	1000/3000	30	
13 (**5b**)	*o*-DCB	20	3000/1000	30	

**Table 2:** Gradients used at the flow of 1 mL min^−1^ for the separation of the compounds on the column.

Time (min)	A (%)	B (%)
0.00	100.0	0.0
2.00	100.0	0.0
10.00	5.0	95.0
21.00	5.0	95.0
22.00	100.0	0.0
28.00	100.0	0.0

**Table 3:** Estimated values of HOMO/LUMO levels and optical bandgaps for each compound.

Compound	Cyclic Voltammetry			UV-VIS Spectroscopy	
	HOMO (eV)	LUMO (eV)	E_g_ (eV)	E_g_ (eV)	LUMO (eV) *
**4b**	−5.07	-	-	1.65	−3.42
**5b**	−5.19	-	-	1.67	−3.52
**5c**	−5.34	−3.68	1.66	1.66	−3.68

* Calculated from HOMO_CV_ and the optical band gap.

## Data Availability

The data presented in this study are available on request from the corresponding author.
